# Intravenous Ferric Carboxymaltose Versus Oral Ferrous Sulfate for Iron Deficiency Anemia: A Retrospective Cohort Study of Iron Store Repletion and Safety

**DOI:** 10.7759/cureus.112040

**Published:** 2026-07-04

**Authors:** Yasmine El Ouafa, Inasse Mourabiti, Nabiha El Abdi, Monsif Fadi, Houda Benrahma, Nouama Bouanani

**Affiliations:** 1 Interdisciplinary Laboratory of Biotechnology and Health, Center for Doctoral Studies (CeDoc), University Mohammed VI of Health Sciences (UM6SS), Casablanca, MAR; 2 Department of Hematology, University Mohammed VI of Health Sciences (UM6SS), Casablanca, MAR; 3 Oncopathology, Cancer Biology and Environment Laboratory, Center for Doctoral Studies (CeDoc), University Mohammed VI of Health Sciences (UM6SS), Casablanca, MAR

**Keywords:** ferritin levels, intravenous iron, iron deficiency anemia (ida), oral iron therapy, retrospective cohort

## Abstract

Background

Iron-deficiency anemia (IDA) is a major clinical burden, particularly when caused by gastrointestinal or gynecological blood loss and mucosal malabsorption. Oral iron supplementation, while widely accessible, is frequently limited by poor gastrointestinal tolerability and inadequate absorption. Intravenous (IV) iron offers a more direct route to iron repletion, yet real-world comparative data from resource-limited settings remain scarce.

Methods

We conducted a retrospective observational cohort study of 225 patients with IDA managed at the Hematology Department of Cheikh Khalifa Hospital, Mohammed VI University of Health Sciences (UM6SS), Casablanca, Morocco, between January 2020 and December 2023. Patients received either oral ferrous sulfate (n=185) or IV ferric carboxymaltose (n=40) based on clinical indication. The primary efficacy endpoint was post-treatment serum ferritin at three months, the only post-treatment laboratory parameter systematically available across both groups. Secondary endpoints included transfusion requirement and adverse event profile.

Results

Both groups were well-matched at baseline across all haematological parameters (hemoglobin (Hb): 8.98±2.23 vs. 8.50±1.42 g/dL, p=0.196; ferritin: 5.57±4.25 vs. 4.71±2.96 µg/L, p=0.221). IV iron was associated with dramatically superior iron store repletion at three months (post-treatment ferritin: 412.60±95.87 vs. 8.68±4.38 µg/L; p<0.001), representing approximately a 47-fold difference. Oral iron recipients remained below the accepted thresholds for adequate store repletion (≥30 µg/L) at follow-up. Transfusion rates were 10.3% (19/185) oral vs. 17.5% (7/40) IV, a non-significant difference (p=0.306), reflecting greater baseline severity in the IV group. Gastrointestinal adverse events occurred in 100% of oral iron recipients, while systemic reactions (predominantly arthralgia, myalgia, and fever) occurred in 50% (20/40) of IV iron recipients; one anaphylactic reaction (1/40; 2.5%) was successfully managed.

Conclusion

In this real-world cohort, IV ferric carboxymaltose achieved markedly superior iron-store repletion compared to oral ferrous sulfate, with a better gastrointestinal tolerability profile, at the cost of a distinct systemic adverse event profile requiring clinical monitoring. These findings support broader consideration of IV iron in patients with IDA who have failed, or are unlikely to benefit from, oral therapy, particularly those with pre-existing mucosal pathology or ongoing haemorrhagic loss.

## Introduction

Iron deficiency anemia (IDA) is the most common micronutrient deficiency in the world, affecting over 1.6 billion people across all age groups and income levels [1,2]. It is far more than a laboratory finding: the fatigue, cognitive impairment, and reduced exercise tolerance it causes translate into real losses in productivity, reduced quality of life, and, in vulnerable populations, a struggle for survival [3,4].

When IDA originates from the gastrointestinal tract, the clinical picture becomes particularly complex. Chronic blood loss from peptic ulcers, colorectal lesions, or angiodysplasia, combined with iron malabsorption in conditions such as celiac disease or inflammatory bowel disease (IBD), creates a self-perpetuating cycle that is difficult to break [5,6]. Mucosal inflammation not only drives the blood loss but also actively impairs the absorption of dietary and supplemental iron, while hepcidin upregulation further restricts iron availability to erythroid precursors [7,8]. The result is an anemia that is often resistant to standard oral treatment and prone to relapse [9].

Oral iron - typically ferrous sulfate - has been the default first-line treatment for decades, valued primarily for its low cost and ease of prescription [10]. In practice, however, up to 60% of patients experience gastrointestinal side effects significant enough to compromise adherence [10,11]. Even among those who tolerate it, absorption is inconsistent and heavily regulated by hepcidin, leading to slow and incomplete responses that can stretch over many months [12].

Intravenous iron bypasses these barriers entirely. By delivering iron directly into circulation, it produces rapid and consistent rises in hemoglobin and ferritin, independent of gut function or inflammatory status [13]. Multiple randomized trials and meta-analyses have confirmed the superiority of intravenous iron over oral iron in inflammatory bowel disease, chronic kidney disease, perioperative anemia, and heart failure, without a proportional increase in serious adverse events [14-16]. In patients with heart failure and iron deficiency, intravenous ferric carboxymaltose has consistently demonstrated improvements in functional status, exercise capacity, and quality of life, and is recommended by current American College of Cardiology/American Heart Association (ACC/AHA) heart failure guidelines [17,18]. More recent formulations such as ferric carboxymaltose have further simplified administration, allowing large doses to be given in a single infusion and reducing the burden on both patients and healthcare systems [19]. Yet despite this evidence, IV iron remains underused in many clinical settings, particularly in lower-resource environments where infusion infrastructure is limited and cost considerations weigh heavily [20].

There is a genuine need for real-world data that can inform clinicians about when and for whom IV iron offers a meaningful clinical advantage over oral supplementation.

This study addresses that need. We report the outcomes of 225 patients with IDA treated at the Hematology Department of Cheikh Khalifa Hospital, Casablanca, Morocco, comparing the hematologic efficacy, iron store repletion, and safety of IV ferric carboxymaltose versus oral ferrous sulfate over a three-month follow-up period.

## Materials and methods

Study design, setting, and ethics

This was a retrospective observational cohort study conducted at the Hematology Department of Cheikh Khalifa Hospital, Mohammed VI University of Health Sciences (UM6SS) in Casablanca, Morocco. We reviewed the records of consecutive patients diagnosed with IDA between January 2020 and December 2023. The study was approved by the Institutional Ethics Committee of UM6SS (approval number: CE/UM6SS/34/24 dated July 23, 2024). As the study was retrospective and all data were fully anonymized, the requirement for individual informed consent was waived in accordance with national regulations. All procedures adhered to the principles of the Declaration of Helsinki.

Patient selection

We included adult patients (aged ≥18 years) with a confirmed diagnosis of IDA of gastrointestinal origin. IDA was defined as hemoglobin below gender-specific thresholds (<12 g/dL in women, <13 g/dL in men), accompanied by microcytic hypochromic indices (mean corpuscular volume (MCV) <80 fL and/or mean corpuscular hemoglobin concentration (MCHC) <32 g/dL) and serum ferritin below 30 ng/mL. A gastrointestinal etiology was established through clinical assessment, endoscopy, and/or imaging.

We excluded patients with anemia attributable to other causes (chronic kidney disease, hematologic malignancies, hemolytic anemia, vitamin B12, or folate deficiency), those who had received a blood transfusion within the 30 days prior to inclusion, and those with incomplete pre- or post-treatment laboratory data.

Treatment allocation and dosing

Treatment was allocated based on the clinical judgment of the attending gastroenterologist and was not randomized. During the study period (2020-2023), oral ferrous sulfate (200 mg per tablet, equivalent to 65 mg elemental iron, administered two to three times daily according to local institutional practice) was prescribed as first-line therapy in patients without contraindications. We acknowledge that more recent evidence and contemporary guidelines increasingly favor lower-dose once-daily or alternate-day oral iron regimens to improve absorption and gastrointestinal tolerability [12]. IV ferric carboxymaltose (Ferinject®) was reserved for patients meeting one or more of the following criteria: documented intolerance or prior failure of oral iron; severe or rapidly symptomatic anemia requiring prompt correction; significant malabsorption (post-bariatric surgery, active celiac disease); or active inflammatory bowel disease.

For patients receiving IV iron, the total iron deficit was calculated using the Ganzoni formula [21]:

\[\text{Total Iron Deficit (mg)} = \text{Body Weight (kg)} \times \left[\text{Target Hb (g/dL)} - \text{Actual Hb (g/dL)}\right]\times 2.4 + 500\]

where Hb represents hemoglobin concentration (g/dL) and 500 mg corresponds to the estimated iron stores required for repletion.

Target hemoglobin was set at 12 g/dL for women and 13 g/dL for men. Ferric carboxymaltose was infused at up to 1000 mg per session (15 mg/kg, maximum 1000 mg) over at least 15 minutes, with a repeat infusion after one week where the calculated deficit exceeded 1000 mg. All infusions were carried out under direct medical supervision, with a mandatory 30-minute post-infusion observation period and immediate access to resuscitation equipment and epinephrine.

Data collection and outcomes

Data were extracted from electronic medical records and, where necessary, paper charts. We collected demographic information (age, sex), clinical presentation (symptoms, bleeding source, behavioral disturbances such as Pica syndrome (persistent craving and consumption of non-food substances)), results of digestive workup (upper and lower endoscopy, abdominal ultrasound), comorbidities, treatment details, and adverse events. Laboratory values - hemoglobin, MCV, MCHC, and serum ferritin - were recorded at baseline and at three months post-treatment.

The primary efficacy endpoint was post-treatment ferritin at three months, the only laboratory parameter systematically available across both treatment groups. Changes in Hb, MCV, and MCHC were explored when available but were not sufficiently complete for formal comparative analysis. Secondary endpoints were the need for blood transfusion during follow-up, and the adverse event profile. Adverse events were extracted from clinical notes and classified as gastrointestinal (constipation, nausea, bloating, metallic taste) or systemic (arthralgia, myalgia, fever, hypersensitivity reactions), with severity graded as mild, moderate, or severe based on the clinician’s documentation.

Statistical analysis

Continuous variables are reported as mean±standard deviation. Categorical variables are expressed as counts and percentages. Group comparisons used Student's t-test for continuous variables and Fisher's exact test or the chi-square test for categorical variables, as appropriate. A p-value below 0.05 was considered statistically significant. All analyses were performed in Python 3.10 (Python Software Foundation, Fredericksburg, VA) using the SciPy 1.9 and pandas 1.5 libraries.

## Results

Patient characteristics

Our cohort included 225 patients with IDA referred to a tertiary hematology department, of whom 185 (82.3%) received oral iron supplementation and 40 (17.7%) received intravenous (IV) ferric carboxymaltose. Women constituted 88.0% (n=198/225) of the cohort, reflecting the predominance of iron deficiency related to gynaecological blood loss in this population. The mean age of the subjects was 38.0±17.6 years, reflecting the broad etiological spectrum encountered across age groups (Table 1).

**Table 1:** General characteristics of the study population SD: standard deviation. Student’s t-test for continuous variables; Chi-squared test for categorical variables. p<0.05 considered statistically significant.

Variable	Oral iron (n=185)	Injectable iron (n=40)	Total (n=225)	p-value
Sex				
Female: n (%)	160 (86.5%)	38 (95.0%)	198 (88.0%)	0.217
Male: n (%)	25 (13.5%)	2 (5.0%)	27 (12.0%)	-
Mean age±SD (years)	38.5±18.3	35.8±13.8	38.0±17.6	0.381
Age distribution: n (%)				
<20 years	N/D	N/D	25 (11.1%)	-
20-40 years	N/D	N/D	117 (52.0%)	-
40-60 years	N/D	N/D	52 (23.1%)	-
>60 years	N/D	N/D	31 (13.8%)	-

**Table 2:** Etiologies of iron deficiency Multiple aetiologies were identified in some patients. Percentages are calculated on the total study population (n=225).

Aetiology	n	%
Gynecological blood loss		
Menorrhagia	28	12.4%
Metrorrhagia	8	3.6%
Other genital bleeding	15	6.7%
Uterine fibroid	1	0.5%
Ovarian cyst	3	1.4%
Gastrointestinal blood loss		
Gastritis (including *Helicobacter pylori*)	20	8.9%
Crohn’s disease	4	1.8%
Angiodysplasia	4	1.8%
Tumours	3	1.4%
Haemorrhoidal disease	3	1.4%
Diverticulosis	1	0.5%
Epistaxis	2	0.9%
Malabsorption / Dietary deficiency		
Coeliac disease	4	1.8%
Pica syndrome	30	13.3%
Dietary deficiency (no identifiable blood loss source)	58	25.8%

Fatigue was the most common presenting symptom, reported in 40.9% of patients (n=92), followed by weight loss in 6.2% (n=14), headache in 6.7% (n=15), exertional dyspnoea in 4.4% (n=10), and vertigo in 2.7% (n=6). Gynecological blood loss was identified as a contributing aetiology in 36 patients: Menorrhagia was identified in 28 patients (12.4%) and metrorrhagia in eight (3.6%) patients. Additional gynecological causes included other genital bleeding (n=15), uterine fibroid (n=1), and ovarian cysts (n=3). Pica syndrome was noted in 13.3% of patients (n=30) - an often underrecognized behavioral manifestation of severe iron deficiency - and confirmed coeliac disease contributed to malabsorption in 1.8% (n=4). Upper GI endoscopy was performed in 24 patients and identified gastric pathology (gastritis, *Helicobacter pylori* infection, or polyps) in 19 cases. Structural abnormalities - uterine fibroids, hemorrhagic ovarian cysts, or colonic diverticulosis - were identified by abdominal imaging in 11 additional patients (Table 2).

The two treatment groups were statistically comparable at baseline across all hematological parameters: hemoglobin (9.0±2.2 vs. 8.5±1.4 g/dL, p=0.196), MCV (70.8 ± 8.7 vs. 70.3 ± 8.5 fL, p=0.729), MCHC (29.2±4.0 vs. 28.7±2.4 g/dL, p=0.453), and serum ferritin (5.6±4.3 vs. 4.7±3.0 μg/L, p=0.221). Menorrhagia was present in 10.8% of the oral iron group (n=20/185) and 20.0% of the IV iron group (n=8/40), a difference that did not reach statistical significance (p=0.183). Metrorrhagia was present in 2.2% (4/185) vs 10.0% (4/40), respectively. Collectively, active gynecological bleeding was numerically more frequent in the IV iron group, likely reflecting the clinical triage pattern whereby physicians preferentially directed patients with active and treatment-resistant bleeding toward parenteral therapy (Table 3).

**Table 3:** Baseline hematological parameters before treatment MCV: mean corpuscular volume. MCHC: mean corpuscular hemoglobin concentration. SD: standard deviation. No statistically significant difference between groups at baseline (all p>0.05), confirming between-group comparability.

Parameter	Oral iron (n=185) (mean±SD)	Injectable iron (n=40) (mean±SD)	Overall (n=225)	p-value
Hemoglobin (g/dL)	9.0±2.2	8.5±1.4	8.9±2.1	0.196
MCV (fL)	70.8±8.7	70.3±8.5	70.7±8.7	0.729
MCHC (g/dL)	29.2±4.0	28.7±2.4	29.1±3.8	0.453
Ferritin (μg/L)	5.6±4.3	4.7±3.0	5.3±4.0	0.221
Haemoglobin distribution: n (%)				
<4 g/dL	N/D	N/D	2 (0.9%)	-
4-6 g/dL	N/D	N/D	19 (8.4%)	-
6-8 g/dL	N/D	N/D	56 (24.9%)	-
8-10 g/dL	N/D	N/D	83 (36.9%)	-
≥ 10 g/dL	N/D	N/D	65 (28.9%)	-
Erythrocyte indices				
Microcytosis (MCV <80 fL): n (%)	-	-	203 (90.2%)	-
Hypochromia (MCHC <32 g/dL): n (%)	-	-	186 (82.7%)	-

Primary hematologic outcomes

Post-treatment hemoglobin, MCV, and MCHC values were not systematically captured in the dataset at a standardized follow-up interval, precluding the calculation of mean ΔHb or recovery of red cell indices. This is acknowledged as a key limitation (see Limitations). The principal efficacy endpoint available for between-group comparison is post-treatment serum ferritin, which was recorded for all 225 patients.

The ferritin response diverged dramatically between treatment modalities. Patients receiving IV ferric carboxymaltose achieved a mean post-treatment ferritin of 412.6±95.9 μg/L, compared with 8.7±4.4 μg/L in the oral iron group (Table 4), a statistically highly significant difference (p<0.001) representing approximately a 47-fold greater iron store repletion. Patients completing three months of oral iron therapy remained with ferritin values well below any established threshold for adequate store repletion (commonly defined as ≥30-50 μg/L), placing them at a substantial risk of symptomatic relapse, particularly in the context of ongoing gynecological blood loss or impaired mucosal absorption. In contrast, the IV iron group achieved ferritin levels indicative of complete and sustained iron store repletion (Table 4).

**Table 4:** Post-treatment ferritin, transfusion rate and clinical outcome SD: standard deviation. Student’s t-test for ferritin comparison. Chi-squared test for transfusion rate. The highly significant difference in post-treatment ferritin (p<0.001) reflects the pharmacokinetic superiority of injectable iron in rapidly replenishing iron stores.

Parameter	Oral iron (n=185)	Injectable iron (n=40)	p-value
Ferritin before treatment (μg/L): mean±SD	5.6±4.3	4.7±3.0	0.221
Ferritin after treatment (μg/L): mean±SD	8.7±4.4	412.6±95.9	<0.001
Blood transfusion: n (%)	19 (10.3%)	7 (17.5%)	0.306
Clinical outcome (overall n=225)			
Resolution of anemia syndrome	N/D	N/D	178 (79.0%)
Stabilization	N/D	N/D	34 (15.0%)
Worsening	N/D	N/D	13 (6.0%)

Overall clinical outcome data, available from physician records, showed the resolution of the anemia syndrome in 79.0% (178/225) of all patients, stabilization in 15.0% (34/225), and worsening in 6.0% (13/225). Between-group outcome data at the individual level were not available for disaggregation.

Transfusion requirement

Twenty-six patients (11.6%) (26/225) required blood transfusion during the follow-up period: 19 in the oral iron group (10.3%) and seven in the IV iron group (17.5%). This difference did not reach statistical significance (p=0.306). The numerically higher transfusion rate among IV iron recipients almost certainly reflects the greater clinical severity and complexity of disease in this group, since patients were allocated to parenteral therapy precisely because their anemia was more acute, refractory, or associated with active ongoing hemorrhage. The study was not powered to detect a difference of this magnitude in transfusion rate.

Adverse events

At least one gastrointestinal symptom was reported in the oral iron group, with a 100% symptom prevalence reflecting the combination of active systematic elicitation at each follow-up visit and the high background burden of pre-existing mucosal pathology in this cohort. Constipation alone was the most frequent complaint (50.8%, n=94/185), followed by bloating alone (24.3%, n=45/185), digestive discomfort and epigastric discomfort together accounted for 11.9% (n=22/185), nausea 6.5% (n=12/185), and metallic taste combined with constipation 4.9% (n=9/185). Constipation in any combination (alone or with another symptom) was present in 57.3% (n=106/185) of patients. No patient was entirely symptom-free. No discontinuation due to adverse events was documented in available clinical records.

We acknowledge that a 100% adverse event rate exceeds the 30%-60% rate typically reported in the retrospective literature. This figure reflects two converging factors: (i) the high background prevalence of pre-existing gastric and mucosal pathology in our population (gastritis or *H. pylori* infection confirmed endoscopically in 19 patients, Crohn’s disease in four, angiodysplasia in four), and (ii) the systematic, active symptom elicitation conducted at every follow-up visit - a design approach that captures complaints that would otherwise go unreported in retrospective case series. No standardized toxicity grading scale (e.g., Common Terminology Criteria for Adverse Events (CTCAE)) was applied; severity was documented as recorded by the treating physician.

The experience with IV ferric carboxymaltose was markedly different in character. Half of IV iron recipients (50.0%, n=20) reported no adverse events. Among those who experienced reactions, arthralgia was the most common (20.0%, n=8), followed by myalgia (15.0%, n=6), fever (15.0%, n=6), and pruritus (5.0%, n=2). One case of anaphylaxis was observed; this reaction was promptly managed with epinephrine and corticosteroids, without lasting sequelae. One additional serious event - a pulmonary embolism occurring on day 14 post-infusion in a patient with established independent thromboembolic risk factors (obesity, prolonged immobility) - was recorded and is discussed in the limitations (Table 5).

**Table 5:** Adverse events by route of administration The 100% rate of GI adverse events in the oral iron group reflects systematic and active questioning at each follow-up visit (prospective collection), as opposed to spontaneous reporting used in retrospective studies. Chi-squared test: p<0.001 for comparison of any adverse event (oral vs. injectable).

Adverse event	Oral iron (n=185): n (%)	Injectable iron (n=40): n (%)
Gastrointestinal adverse events (oral iron)		
Constipation (alone)	94 (50.8%)	-
Bloating (alone)	45 (24.3%)	-
Digestive discomfort	20 (10.8%)	-
Metallic taste + constipation	9 (4.9%)	-
Nausea + bloating	6 (3.2%)	-
Nausea (alone)	6 (3.2%)	-
Constipation+bloating	3 (1.6%)	-
Epigastric discomfort	2 (1.1%)	-
Total gastrointestinal (GI) adverse events	185 (100%)*	0 (0%)
Systemic adverse events (injectable iron): p<0.001 vs. oral iron		
None (no adverse event)	-	20 (50.0%)
Arthralgia	-	8 (20.0%)
Myalgia	-	6 (15.0%)
Fever	-	6 (15.0%)
Pruritus/skin reaction	-	2 (5.0%)
Anaphylactic shock	—	1 (2.5%)
Pulmonary embolism	—	1 (2.5%)
Total with adverse events	—	20 (50.0%)

## Discussion

The principal finding of this study is that IV ferric carboxymaltose achieves markedly superior iron store repletion compared with oral iron in patients with IDA managed in a tertiary hematology setting. The post-treatment ferritin difference - 412.60±95.87 vs. 8.68±4.38 μg/L (p<0.001) - is not merely statistical: it represents two fundamentally different biological outcomes. Patients completing oral therapy remained with ferritin levels that would widely be considered inadequate for sustained clinical remission, while patients receiving IV iron achieved full store repletion. At the same time, IV iron was far better tolerated from a gastrointestinal perspective, even as it carried its own systemic adverse event profile that was largely mild and transient.

These findings are consistent with the broader literature. In the FERGIcor randomized controlled trial, Evstatiev et al. reported a mean hemoglobin advantage of 0.9-1.5 g/dL favouring IV iron [22]. Bonovas et al. confirmed in a separate meta-analysis that IV iron yields superior haematologic responses without a proportional rise in serious adverse events [23]. Our ferritin data are particularly striking: a 47-fold difference in iron store repletion at three months is consistent with the known pharmacokinetic superiority of IV formulations, which bypass intestinal absorption entirely and deliver iron directly to hepatic stores and bone marrow [20].

Current international recommendations support the use of IV iron as first-line therapy in patients with IBD, intolerance to oral iron, or when rapid iron repletion is required [24,25].

The 100% gastrointestinal adverse event rate in the oral iron group warrants methodological clarification. This figure reflects two converging phenomena: the high background burden of pre-existing mucosal pathology in our cohort and the systematic, active symptom elicitation at every follow-up visit. Published prospective data similarly report GI adverse event rates of 50%-70% with ferrous sulfate when patients are questioned in detail [10,11] - substantially higher than the 30-60% cited in retrospective series where only spontaneously reported events are captured. The adverse event profile in our population reinforces the clinical case for replacing ferrous sulfate with better-tolerated formulations - ferric maltol or liposomal iron - in patients with established GI vulnerability [26,27]. It should also be acknowledged that the oral iron regimen used in this retrospective cohort reflected local institutional practice during the study period. Emerging evidence now supports lower-dose once-daily or alternate-day oral iron schedules, which improve fractional iron absorption through reduced hepcidin induction and are associated with better gastrointestinal tolerability [12,24]

Regarding IV iron safety, the 50% overall systemic adverse event rate driven by arthralgia (20.0%, n=8/40), myalgia (15.0%, n=6/40), and fever (15.0%, n=6/40) is consistent with the known pharmacological profile of ferric carboxymaltose. Although numerically higher than the 0.5%-1.5% rates generally reported in the IV iron literature, this difference is likely explained by the small size of the IV cohort [28]. The pulmonary embolism occurring at day 14 post-infusion in a patient with multiple independent thromboembolic risk factors cannot be attributed causally to ferric carboxymaltose on current evidence [29]; no mechanistic link has been established in the literature. These reactions reflect the phenomenon of complement activation-related pseudo-allergy (CARPA) [30] and were predominantly grade 1-2, short-lived, and manageable without treatment discontinuation. A single patient in the intravenous ferric carboxymaltose group experienced an anaphylactic reaction (2.5%, 1/40), which was managed promptly without further complications.

The numerical imbalance in gynecological blood loss between groups (menorrhagia: 10.8% (20/185) vs. 20.0% (8/40); metrorrhagia: 2.2% (4/185) vs. 10.0% (4/40)) did not reach statistical significance (p=0.183) but reflects a clinically important triage pattern: patients with active, treatment-resistant hemorrhage were appropriately directed toward parenteral therapy. This channelling bias, well-recognized in comparative effectiveness research [31], means the IV iron group harbored intrinsically harder-to-correct iron deficits, and yet still achieved dramatically superior ferritin repletion. Future studies should stratify analyses by bleeding aetiology to fully disentangle pharmacological from etiological effects.

The transfusion rate comparison (oral 10.3% vs. IV 17.5%, p = 0.306) is underpowered and should not be over-interpreted in either direction. The numerical trend toward higher transfusion in the IV group reflects disease severity at baseline rather than any adverse pharmacological effect. As with all retrospective observational studies, our design is subject to inherent limitations, including selection bias and confounding, even though such designs have been shown to produce results consistent with randomized trials in many clinical contexts [32]. From a health economics standpoint, each avoided transfusion represents meaningful savings in direct costs, infectious risk, and nursing burden, reinforcing the economic case for appropriately targeted IV iron use [33].

Network meta-analyses further suggest that ferric carboxymaltose and ferric derisomaltose offer comparable efficacy among available IV formulations, with no significant differences in serious adverse events [34].

## Conclusions

In this cohort of 225 real-world patients with IDA, IV ferric carboxymaltose achieved dramatically superior iron store repletion compared to oral ferrous sulfate (post-treatment ferritin 412.60±95.87 vs. 8.68±4.38 μg/L; p<0.001), with markedly better gastrointestinal tolerability, at the cost of a distinct systemic adverse event profile requiring clinical monitoring. These findings support broader consideration of IV iron in patients with IDA who have failed, or are unlikely to benefit from, oral therapy - particularly those with pre-existing mucosal pathology, ongoing hemorrhagic loss, or documented oral intolerance. Prospective studies with standardized post-treatment hematological assessment, patient-reported outcomes, and formal cost-effectiveness analyses are needed to fully establish the place of IV iron in routine clinical practice in resource-limited settings.
